# Lack of association between nutritional status and change in clinical category among HIV-infected children in Brazil

**DOI:** 10.1590/S1516-31802005000200006

**Published:** 2005-03-02

**Authors:** Maraisa Centeville, André Moreno Morcillo, Antonio de Azevedo Barros, Marcos Tadeu Nolasco da Silva, Adyléia Aparecida Dalbo Contrera Toro, Maria Marluce dos Santos Vilela

**Keywords:** HIV, Acquired immunodeficiency syndrome, Child, Nutritional status, Nutrition, HIV, Síndrome de imunodeficiência adquirida, Criança, Nutrição, Estado nutricional

## Abstract

**CONTEXT AND OBJECTIVE:**

Malnutrition is common among HIV-infected children. Our objective was to study the occurrence of malnutrition and its relationship with changes in clinical category among HIV-infected children.

**DESIGN AND SETTING:**

Longitudinal study, at the Pediatrics Department and Pediatrics Investigation Center (CIPED), Faculdade de Ciências Médicas da Universidade Estadual de Campinas (Unicamp).

**METHODS:**

We reviewed the hospital records of 127 vertically HIV-infected children. Anthropo-metric measurements were obtained at the beginning of follow-up, at clinical category change and five months later. These were converted to z-scores of weight/age, height/age and weight/height. Data were presented as means, standard deviations, frequency counts and percentages. The Wilcoxon and Kruskal-Wallis tests and odds ratios were used in the analysis.

**RESULTS:**

We found that 51 (40.2%) were undernourished and 40 (31.5%) were stunted, with higher risk of being included in clinical category C. There was an association between nutritional condition and the clinical categories of the Centers for Disease Control classification (1994), and with age at symptom onset (except for height z-score). During follow-up, 36 patients (28.4%) changed their clinical category, which occurred early among the undernourished patients. The group that changed its clinical category maintained the same z-score distribution for weight, height and weight/height throughout follow-up.

**CONCLUSION:**

Aids manifestation severity was associated with nutritional status and with age at symptom onset, but change in clinical category was not followed by worsening of nutritional status.

## RESUMO

**CONTEXTO E OBJETIVO:**

A ocorrência de desnutrição é freqüente em crianças com infecção pelo HIV. O objetivo do estudo foi estudar a ocorrência de desnutrição e sua relação com a mudança de categoria clínica em crianças infectadas pelo HIV.

**TIPO DE ESTUDO E LOCAL:**

Estudo longitudinal, no Departamento de Pediatria e Centro de Investigação em Pediatria (CIPED). Faculdade de Ciências Médicas da Universidade Estadual de Campinas (Unicamp).

**MÉTODOS:**

Revisamos os prontuários de 127 pacientes com infecção perinatal pelo HIV com o propósito de obter medidas de peso e estatura no início do acompanhamento ambulatorial, no momento da mudança de categoria clínica e cinco meses após a mudança. Estes dados foram transformados em z-escores de peso/idade, altura/idade e peso/altura. Os testes de Wilcoxon, Kruskal-Wallis e o cálculo da razão de chances foram usados.

**RESULTADOS:**

51 (40,2%) das crianças avaliadas apresentavam desnutrição, sendo 40 (31,5%) com comprometimento de altura, e portanto com maior risco de inclusão na categoria clínica C. Encontramos associação entre condição nutricional, categoria clínica e idade no início dos sintomas. 36 (28,4%) pacientes mudaram de categoria clínica durante o acompanhamento, e a mudança ocorreu em idade mais precoce entre os desnutridos. O grupo que mudou de categoria clínica manteve as mesmas distribuições de z-escore de peso, altura e peso/altura durante o acompanhamento.

**CONCLUSÃO:**

A gravidade das manifestações da aids associa-se com a condição nutricional e com a idade de início dos sintomas. A mudança de categoria clínica não se acompanhou de piora no estado nutricional.

## INTRODUCTION

Acquired immunodeficiency syndrome (Aids) is associated with different levels of nutritional deficiency among adults and children, whose condition takes on different characteristics and evolution.^1^ Even compared with seroreverter children exposed to the same social conditions, the growth of infected children is severely affected.^2^

In the pediatric Aids classification (Centers for Disease Control and Prevention, CDC, 1994),^3^ clinical category N refers to patients without symptoms, category A mild symptoms, category B moderate symptoms and category C severe manifestations related to opportunistic diseases.

The occurrence of wasting syndrome and failure to thrive place the child in category C. The Brazilian Ministry of Health has also adopted the same classification criteria.^4^ This classification, however, does not consider mild degrees of undernutrition, although weight loss is one of the initial symptoms for this diagnosis.

Nutritional status, including growth, is influenced by morbidity and mortality of the disease.^5,6^ This has been affected beneficially through the introduction of highly active antiretroviral therapy (HAART).^7^

In this paper, our goal was to retrospectively evaluate the distribution of weight and height z-scores among children infected by HIV-1, correlate the findings with the clinical classification and also investigate the association between malnutrition and changes in clinical category during the evolution of the disease.

## METHODS

**Type of study:** This was a longitudinal retrospective study.

**Sample and setting:** From a cohort of 147 children in the metropolitan region of Campinas, State of São Paulo, who were referred to the Aids children division of Universidade Estadual de Campinas university hospital between January 1989 and July 1999, we selected a total of 127 vertically infected children. These infants received medical care in the outpatient service at least once a month on a routine basis. Of these 127 selected children, 71 were males (55.9%) and 56 were females (44.1%), and all fulfilled the diagnostic criteria for Aids.^3,4^

**Main measurements:** From each patient's hospital record we obtained information about gender, birth weight, breastfeeding, age at onset of symptoms, antiretroviral therapy, weight, height and CDC clinical category at diagnosis (measurement M1), at the time of category change (M2) and five months after that change (M3). The clinical categories N and A were analyzed together.

According to their ages at disease onset, the children were grouped as rapid progressors (signs before 12 months old) or slow progressors (signs after 12 months old).

Weight was measured using a Filizola electronic balance. Up to the age of two years, the length was measured with a horizontal wooden anthropometer and, after this age, with a vertical one.

Waterlow et al.^8^ proposed a functional classification for child malnutrition that separated children who had acute malnutrition from those with chronic malnutrition. The acutely malnourished children were those with adequate height for age but inadequate weight for height (wasted). The chronically malnourished children were those that had inadequate height for age (stunted). Chronically malnourished children could also be acutely malnourished, in which case they would be both stunted and wasted.

**Statistical methods:** The weight and height values were transformed into z-scores, utilizing National Center for Health Statistics (NCHS)^9^ as the reference curve. The Waterlow classification was used to determine the nutritional status, taking z scores of less than −2 standard deviations (SD) as the cutoff points.^8^ Z-scores outside of these parameters were not included.

Descriptive data were reported as means with standard deviations, frequency counts and percentages. For statistical analysis the Wilcoxon and Kruskal-Wallis tests and odds ratios were used.

## RESULTS

The general characteristics of the children are shown in Table 1. At admission, 54 (42.5%) were classified in clinical category A or N, 58 (45.7%) in category B and 15 (11.8%) in category C.

**Table 1:** General characteristics of HIV-1 vertically infected children at diagnosis Characteristics (number of patients)

Characteristics (number of patients)		
Age at onset of symptoms (108)	Mean (months)	18.96
	Median (months)	9
Gender (127)	Male	71 (55.9%)
	Female	56 (44.1%)
Clinical Category (127)	N/A no or mild symptoms	54 (42.5%)
	B moderate symptoms	58 (45.7%)
	C severe symptoms	15 (11.8%)
Nutrition (119)	Eutrophic	68 (57.1%)
	Stunted	40 (33.7%)
	Chronic malnourished	8 (6.7%)
	Wasted	3 (2.5%)
Clinical Category Change (127)	Yes	36 (28.3%)
	No	91 (71.7%)
Outcome (127)	Death	29 (22.8%)
	Survival	98 (77.2%)

Concerning the children's nutritional status, 68 (53.5%) were classified as eutrophic, 40 (31.5%) as stunted, 8 (6.3%) as chronically undernourished and 3 (2.4%) as wasted. Eight children could not be classified because of incomplete data. The distribution of the z-scores for weight-for-age (p < 0.01), height-for-age (p = 0.002) and weight-for-height (p = 0.01), according to the clinical category at M1, showed statistically significant differences (Table 2).

**Table 2:** Comparison of the mean weight, height and weight/height z-scores of 127 vertically HIV-1 infected children, in clinical categories N, A, B and C at diagnosis

Z-scores	Clinical category	n	Mean	Standard deviation	p-values (Kruskal-Wallis)
Weight	N/A no or mild symptoms	54	-0.97	1.16	<0.001
	B moderate symptoms	58	-0.73	1.61	
	C severe symptoms	15	-2.72	1.02	
Height	N/A no or mild symptoms	54	-1.27	1.07	0.002
	B moderate symptoms	58	-1.77	1.81	
	C severe symptoms	15	-2.63	1.02	
Weight/height	N/A no or mild symptoms	54	-0.007	1.12	0.012
	B moderate symptoms	58	-0.65	1.20	
	C severe symptoms	15	-1.45	1.47	

The mean age at onset of symptoms was recorded for only 108 of the HIV-infected children. It was significantly different between the eutrophic and undernourished groups (p = 0.002). In the eutrophic group, the mean age at onset of the symptoms was 24.19 months (minimum = 1, maximum = 127 and median = 16 months) (Table 3). In this group, height-forage was −1.95 (0.84), weight-for-age was −0.53 (0.97), and weight-for-height was 0.03 (1.05). In the undernourished group, the mean age at onset of symptoms was 13.16 months (minimum = 2, maximum = 86 and median = 7 months) (Table 3). The height-for-age was −2.87 (0.97), weight-for-age was −2.76 (1.01) and weight-for-height was −1.11 (1.21). In this group, the chance of being included in clinical category C was higher than for the eutrophic group (odds ratio = 3.23, confidence interval = 1.40 – 7.53) (Table 4). We found significant associations for weight-for-age (p < 0.05) and weight-for-height (p < 0.05) with age at onset of symptoms, with the lowest values in the rapid progressor group. There was no association for height-for-age (p > 0.05).

**Table 3:** Comparison of the general characteristics between eutrophic and malnourished children at diagnosis of HIV infection

Characteristics (number of cases)		Eutrophic (%)	Malnourished (%)	p-values (Wilcoxon test)
Gender (119)	Male	37 (54.4)	30 (58.8)	
	Female	31 (45.6)	21 (41.2)	
Onset of symptoms in months (108)		24.19	13.16	0.002
Outcome (119)	Death	9 (13.2)	15 (29.4)	
	Survival	59 (86.8)	36 (70.6)	
Time until clinical category change (months)		20	8	0.008
Progression (101)	Rapid	24 (46.2)	37 (72.5)	
	Slow	28 (53.8)	12 (23.5)	
Clinical category (119)	N/A no or mild symptoms	39 (54.7)	15 (29.4)	
	B moderate symptoms	28 (41.2)	27 (52.9)	
	C severe symptoms	1 (1.5)	9 (17.7)	
Clinical category change (119)	Yes	22 (32.4)	14 (27.5)	
	No	46 (67.6)	37 (72.5)	

**Table 4:** Risk for undernourished and eutrophic HIV-infected children to be classified in clinical category C of the Centers for Disease Control and Prevention revised classification system for HIV infection in children, 1994^3^

	n	Category	Categories	OR*	95% CI*
		C = severe symptoms	N = no symptoms A = mild symptoms B = moderate symptoms		
Malnourished	51	36	15	3.23	1.40 – 7.53
Eutrophic	68	29	39		
n	119	65	54		

*OR = odds ratio; CI = confidence interval.*

During follow-up, 36 patients (28.4%), of whom 22 were eutrophic and 14 undernourished, changed their clinical category over a mean period of 20 and 8 months, respectively (p = 0.008). The group that changed clinical category maintained the same weight-for-age, height-for-age and weight-for-height at M1, M2 and M3 (p > 0.05) (Table 5).

**Table 5:** Comparison of the mean weight, height and weight/height z-scores of HIV-1 infected children in clinical categories N, A, B and C* at diagnosis (M1), clinical category change (M2) and 5 months later (M3)

	Measurement (M)	Mean	Standard deviation
Weight	M1	-1.41	1.42
	M2	-1.52	1.69
	M3	-1.51	1.65
Height	M1	-1.62	1.27
	M2	-1.79	1.88
	M3	-1.97	1.97
Weight/height	M1	-0.39	1.06
	M2	-0.49	1.01
	M3	-0.44	1.36

*p > 0.05 (Wilcoxon test).*

* *A/N: mild or no symptoms; B: moderate symptoms; C: severe symptoms.*

## DISCUSSION

In the group of children studied, the occur-rence of malnutrition was associated with the age at onset of symptoms and the severity of CDC clinical category at diagnosis. The clinical category change was earlier among those patients who were already undernourished when diagnosed, although there was no significant change in weight-for-age, height-for-age and weight-for-height for any of the children evaluated.

Our data showed that undernutrition led to a 3.23-times increased risk that the children would be classified in clinical category C (Table 4). Low weight gain and growth failure, which are both severe manifestations of Aids and related to high viral load and low CD4 count,^10,11^ are risk factors that raise the mortality rate among HIV-infected children.^12–14^

The correlation between clinical category and undernutrition reinforces the concept of natural evolution that underlies the CDC classification. This classification system represents the present moment of HIV infection as well as the natural evolution of the disease. The symptoms described in each category follow those that progressively occur within the immunological system among non-treated Aids cases.

The age at which symptoms began was found to be strongly related to nutritional status. The rapid progressors had lower mean weight-for-age and weight-for-height, while the mean age at onset of symptoms was lower among the undernourished children than among the eutrophic children.

Early development of symptoms has been described as a mark of disease severity and decreased survival time.^15,16^ Recently, the use of highly active antiretroviral therapy has improved life expectancy and the quality of life for these children.^17,18^

Progress in the treatment of Aids has changed it into a chronic disease. In fact, the nutritional damage pattern found in the present study, of low weight-for-age and height-for-age with normal weight-for-height, is the same pattern as observed for other chronic diseases.

Malnutrition among HIV-infected patients is multifactorial. Increased energy requirements, decreased calorie intake, intestinal malabsorption and economic problems contribute towards malnutrition among this population.^19,20^

From the outset of follow-up, such patients undergo antiretroviral therapy, opportunistic infection prevention and early treatment for secondary bacterial infections. These actions may result in viral load reduction, improvement of clinical status and less wasting syndrome. Furthermore, social workers play an important role in the overall initiative for Aids therapy by aiding the family with their social, economic and affective problems.

Patient adherence to the overall treatment ensures enhancement of their quality of life. Through this, patients who changed their clinical category during the follow-up may be able to maintain the same initial weight-forage, height-for-age and weight-for-height, in the way that was observed in our study.

## CONCLUSION

We observed that the severity of Aids manifestations was associated with nutritional status and with the age at onset of symptoms, but changes in clinical category were not followed by worsening of the nutritional status.
